# Neuropeptidergic Control of Feeding: Focus on the Galanin Family of Peptides

**DOI:** 10.3390/ijms22052544

**Published:** 2021-03-03

**Authors:** P. Marcos, R. Coveñas

**Affiliations:** 1 Cellular Neuroanatomy and Molecular Chemistry of Central Nervous System, Faculty of Medicine, University of Castilla-La Mancha, CRIB (Regional Centre of Biomedical Research), Avenida de Almansa 14, 02006 Albacete, Spain; 2 Laboratory of Neuroanatomy of the Peptidergic Systems (Group GIR-BMD), Institute of Neurosciences of Castilla y León (INCYL), Laboratory 14, University of Salamanca, c/Pintor Fernando Gallego 1, 37007 Salamanca, Spain; covenas@usal.es

**Keywords:** alarin, celastrol, daidzein, GAL-like peptide, GAL message-associated peptide, GAL receptor, obesity, orexigenic peptides, spexin

## Abstract

Obesity/overweight are important health problems due to metabolic complications. Dysregulation of peptides exerting orexigenic/anorexigenic effects must be investigated in-depth to understand the mechanisms involved in feeding behaviour. One of the most important and studied orexigenic peptides is galanin (GAL). The aim of this review is to update the mechanisms of action and physiological roles played by the GAL family of peptides (GAL, GAL-like peptide, GAL message-associated peptide, alarin) in the control of food intake and to review the involvement of these peptides in metabolic diseases and food intake disorders in experimental animal models and humans. The interaction between GAL and NPY in feeding and energy metabolism, the relationships between GAL and other substances involved in food intake mechanisms, the potential pharmacological strategies to treat food intake disorders and obesity and the possible clinical applications will be mentioned and discussed. Some research lines are suggested to be developed in the future, such as studies focused on GAL receptor/neuropeptide Y Y_1_ receptor interactions in hypothalamic and extra-hypothalamic nuclei and sexual differences regarding the expression of GAL in feeding behaviour. It is also important to study the possible GAL resistance in obese individuals to better understand the molecular mechanisms by which GAL regulates insulin/glucose metabolism. GAL does not exert a pivotal role in weight regulation and food intake, but this role is crucial in fat intake and also exerts an important action by regulating the activity of other key compounds under conditions of stress/altered diet.

## 1. Introduction

The disturbance in the balance between energy intake and energy requirements leads to changes in metabolism. Excess energy is stored as fat, which can be used when food is scarce, but excessive fat accumulation is defined as obesity [1]. Currently, obesity and overweight are important health problems, considered as a growing epidemic, because they can generate some metabolic complications (e.g., type 2 diabetes mellitus, hypertension, coronary artery disease, elevated cholesterol, hyperlipidemia) [2,3]. Obesity also decreases fertility and increases the risks of miscarriage and health problems for mother and child during pregnancy and after birth [4]. A strong association has been found between suboptimal fetal and neonatal nutrition and a number of chronic metabolic conditions later in life, including cardiovascular diseases, hypertension and diabetes [5]. Bariatric surgery is an effective anti-obesity therapy but it is invasive and irreversible. Unfortunately, a therapeutical strategy to treat obesity is not currently available and in some cases, anti-obesity treatments promote serious side-effects. Thus, it is crucial to understand in-depth the mechanisms involved in obesity/overweight to prevent/treat them as well as the associated metabolic complications.

**Figure 1:**
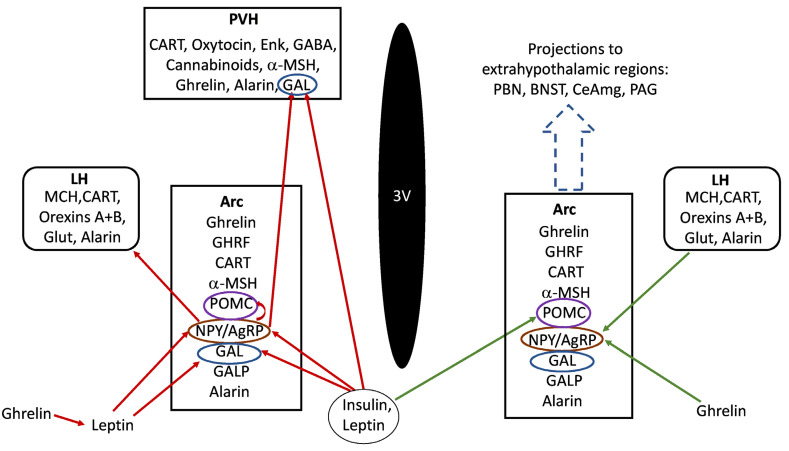
General overview of the most important interactions between the hypothalamic nuclei involved in the control of food intake. The most studied neurochemical substances related to these mechanisms are included in the corresponding hypothalamic nucleus. Green arrows (right side of the figure) represent activation; red arrows (left side of the figure) represent inhibition. Connections of the Arc with other extrahypothalamic areas implicated in the control of feeding behavior are represented by a discontinuous arrow. Abbreviations: α-MSH: alpha-melanocyte stimulating hormone; 3V: third ventricle; Arc: arcuate hypothalamic nucleus; BNST: bed nucleus of the stria terminalis; CART: cocaine- and amphetamine-regulated transcript peptide; CeAmg: central nucleus of the amygdala; Enk: enkephalin; GABA: gamma-aminobutyric acid; GAL: galanin; GALP: galanin-like peptide; GHRF: growth hormone releasing factor; Glut: glutamate; LH: lateral hypothalamus; MCH: melanin-concentrating hormone; NPY/AgRP: neuropeptide Y/agouti-related protein neurons; PAG: periaqueductal gray matter; PBN: parabrachial nucleus; POMC: pro-opio-melanocortin; PVH: paraventricular hypothalamic nucleus.

It is well known that the hypothalamus maintains body weight homeostasis by effectively adjusting food intake and energy expenditure, and contains neuronal groups implicated in the regulation of energy balance [1]. The most important are located in the arcuate (Arc) and paraventricular hypothalamic (PVH) nuclei, although other regions such are the dorsomedial and ventromedial nuclei or the perifornical lateral hypothalamus also participate in this function [6]. Feeding behavior is regulated by peptidergic transmission within the hypothalamus (Figure 1).

One of the ways to treat or prevent obesity/overweight could be to know better the involvement of peptides in feeding behaviour, since they are critical molecules that stimulate (exerting an orexigenic effect) or decrease (exerting an anorexigenic effect) appetite [2,6,7,8], and dysregulation in the signalling of these peptides leads to alterations in food intake, promoting obesity or leanness [7]. Neuroactive substances implicated in the control of feeding behavior include neuropeptides (enkephalin, orexin) and classical neurotransmitters such are dopamine, gamma-aminobutyric acid (GABA) or glutamate [6]. Due to their wide range of action, it is crucial to know in detail the neuronal pathways and the neurochemical substances that modulate food intake. In a simplified manner, circulating plasma levels of ghrelin, cholecystokinin (CCK), glucagon-like peptide-1 (GLP-1), leptin, pancreatic polypeptide (PP) and peptide tyrosine tyrosine (PYY), produced by the stomach, the gut, the pancreas and the adipose tissue reach some regions of the central nervous system (CNS) such as the area postrema in the brainstem or the median eminence in the hypothalamus, where the different structures of the blood–brain barrier allow the access of these substances into the CNS. From these regions, substances may easily arrive in other brain nuclei such as the nucleus of the solitary tract in the brainstem or the hypothalamic Arc which are also connected by afferent and efferent projections [3,9]. In the Arc, two subsets of neurons regulate feeding control. One of them expresses orexigenic peptides (neuropeptide Y (NPY) and agouti-related protein (AgRP)) as well as GABA, and the other group of neurons expresses anorexigenic peptides derived from pro-opio-melanocortin (POMC). NPY/AgRP neurons provide a dominant inhibitory tone onto POMC population; these two subsets of Arc neurons constitute a functional unit due to their common target neurons and the melanocortin receptor 4 [3,9,10] and express receptors for insulin and leptin which are the peripheral signals of satiety [1]. AgRP/NPY neurons of the Arc are activated by peripheral hunger signalling mediated by hormones such as ghrelin secreted by the stomach [11], and are inhibited by leptin or corticotropin-releasing hormone (CRH). AgRP neurons are also activated, via a cAMP-dependent pathway, by the hormone asprosin; this mechanism inhibits the anorexigenic signals mediated by POMC neurons and hence asprosin acts as an orexigenic hormone [12]. Moreover, this hormone induces the production of glucose in the liver [12]. In contrast, POMC neurons are activated by leptin, GLP-1 or insulin [3,9,13,14,15,16] (Figure 1). Recent studies [17] reported the role of liraglutide, a GLP-1 agonist, as an inhibitor of AgRP/NPY neurons by GABA-mediated mechanisms. It has been suggested that leptin’s effects on feeding by AgRP neurons are mediated by changes in neuronal firing, and the control of glucose balance exerted by these cells is independent of chemogenetic activation or inhibition [18]. In addition, leptin might influence the sympathetic innervation of the adipose tissue by acting on AgRP and POMC neurons of the Arc [19]. Growth hormone exerts direct trophic effects on these two neuronal populations leading to the formation of axonal projections to other hypothalamic nuclei [20]. A disbalance in the activity of these two subsets of neurons, which are connected to other brain regions, may lead to feeding alterations. In this sense, a review has been focused on the development of the hypothalamic pathways regulating energy balance/food intake [5] and it has been reported that galanin (GAL), enkephalin, orexin, melanin-concentrating hormone and cannabonids are involved in fat intake; NPY and AgRP in carbohydrate intake, and growth hormone-releasing factor and ghrelin in protein intake [6]. Protein/carbohydrate intake is controlled via negative feedback mechanisms, whereas the intake of fat is mediated by a positive feedback process (pre-ingestive); that is, the ingestion of a rich fat diet stimulates the desire for the ingestion of more fat [6]. A post-ingestive negative feedback pathway for fat ingestion (e.g., lipid messenger oleoylethanolamide) also occurs [21]. In this sense, it is known that oleoylethanolamide (an analogue of the endocannabinoid anandamide) exerts an anorexic effect and that it is involved in the peripheral regulation of feeding [21,22,23,24]. The function of the hypothalamic–pituitary–gonadal axis also affects the physiology of eating and hence sex differences may contribute to obesity [4]. Emotional/reinforcement factors are also pivotal in feeding behaviour [6] and extra-hypothalamic regions (e.g., ventral tegmental area, nucleus accumbens, amygdala) play an important role in these factors [6]. Moreover, neuroactive substances exert their actions throughout the hypothalamus affecting different extra-hypothalamic populations of neurons associated with the regulation of energy homeostasis or mediating food reward [10] (Figure 1).

As indicated above, studies on the neural pathways involved in appetite, including neuroactive substances and their receptors, will help to understand the etiology of obesity and to select new potential therapeutic targets [7]. Many studies have been carried out on the involvement of the orexigenic peptide GAL in feeding behaviour, mainly related to fat intake [25]. GAL belongs to the GAL family of peptides as well as GAL-like peptides, alarin and GAL message-associated peptides [26], all of them implicated in the regulation of food intake. The GAL-like peptide was discovered in the porcine hypothalamus, contains 60 amino acids and cells expressing the peptide have mainly been detected in the posterior pituitary and Arc nucleus [2,27], whereas alarin was discovered in human gangliocytes of neuroblastic tumors, contains 25 amino acids and it has been observed in the hypothalamus (e.g., medial preoptic area, Arc), amygdala and locus coeruleus [2,28,29,30]. Due to the important roles played by the GAL family of peptides in feeding behaviour, the aim of this review is to update the knowledge on the involvement of these peptides in this behaviour. It is important to note that most of the currently available data reported in the literature are focused on the actions exerted by GAL. 

## 2. Galanin

GAL, isolated from the pig upper small intestine, contains 29 amino acids and is well conserved among species. In humans, GAL contains 30 amino acids and shares the sequence of the N-terminal fifteen residues found in other mammals, but other parts of the molecule show some differences (e.g., GAL is not C-terminally amidated as occurs in other mammals) [31]. In humans, the gene encoding GAL/GAL message-associated peptide is located in chromosome 11q13.3 and contains five introns and six exons which are translated into a 123 amino precursor, a pre-prohormone containing the signal peptide, GAL and GAL message-associated peptide [31]. The gene encoding GAL-like peptide/alarin is located in the human chromosome 19q13.43 and shows six exons which in humans are translated into a 116 amino precursor, the pre-pro GAL-like peptide that contains the signal peptide and GAL-like peptide [31]. Alarin is an alternate transcript of the GAL-like gene [32]; in humans, the precursor for alarin (49 amino acids) contains the signal peptide, the first five amino acids of the mature GAL-like peptide and nineteen amino acids without homology to other proteins [31].

**Figure 2:**
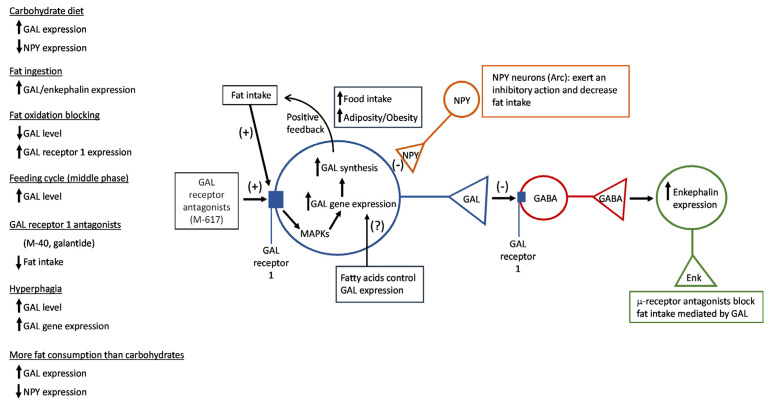
Paraventricular hypothalamic nucleus (PVH): neural pathway and changes in the nucleus after different situations (left side). PVH plays a crucial role in modulating feeding mechanisms. ↑: increase; ↓: decrease; +: activation; -: inhibition; ?: action to be demonstrated. GABA: gamma-aminobutyric acid; GAL: galanin; MAPKs: mitogen-activated protein kinases; NPY: neuropeptide Y.

GAL shows a widespread distribution by the mammalian peripheral and central nervous systems, including the feeding–regulating hypothalamus (e.g., PVH, Arc and dorsomedial nuclei). In addition to feeding, GAL and related peptides are involved in cardiovascular, respiratory, gastrointestinal and neuroendocrine mechanisms, memory, cognition, osmotic/metabolic homeostasis, reproduction, neural growth, arousal, sleep, injury response as well as in depression, anxiety, epilepsy, diabetes mellitus, inflammatory bowel disease, pain, stroke, cancer, alcohol intake, multiple sclerosis, Alzheimer disease (where upregulation of GAL occurs) and affective behaviour [2,26,31]. It has been reported that modifications in pain threshold (antinociceptive and nociceptive) caused by obesity are modulated by GAL [33]. In peripheral regions and organs, cell bodies containing GAL have been detected in the dorsal root ganglia, intramural ganglia and in the paravertebral sympathetic ganglia as well as in neurons located in the myenteric plexus of the gastrointestinal tract. In addition, nerve fibers containing GAL have been detected in the submucous and myenteric plexuses and in sympathetic nerves which innervate the pancreatic islets. Enteric GAL-immunoreactive fibers seem to be of local origin since they are not affected by extrinsic denervation, and it has been demonstrated that GAL induces a concentration-dependent contraction of the gut smooth muscle cells by a direct myogenic effect [34]. The physiological actions mediated by GAL are modulated via GAL 1, 2 and 3 receptors which also show a widespread distribution through the mammalian peripheral and central nervous systems, including the feeding–regulating hypothalamus. Human GAL shows subnanomolar affinity at the GAL 1 receptor, subnanomolar to nanomolar affinity at the GAL 2 receptor and tens of nanomolar affinity at the GAL 3 receptor [34]. Recently, a GAL–GAL receptor signalling network map has been reported that greatly increases the knowledge of the molecules involved in the signalling cascades regulated by the GAL/GAL receptor system [35]. GAL receptors, with seven transmembrane-domains, are G protein-coupled receptors: GAL 1 receptor is coupled to Gα_i_ and/or Gβγ signalling pathways and mediates the activation of mitogen-activated protein kinases (MAPKs) [31] (Figure 2).

In fact, GAL 1 receptor mediates adipogenesis by stimulating MAPK1/MAPK3 activity, via pertussis toxin-sensitive G_α_ inhibitory-subunits, in a Ras/Raf-dependent manner [35]. Moreover, the activation of the GAL 1 receptor is related to the production of cyclic adenosine monophosphate (cAMP) and adenylyl cyclase [35] and improves insulin resistance by inhibiting C-reactive protein release and by favouring the AKT/AS160 cascade [35]. GAL 2 receptor is coupled to the Gα_q/11_ signalling pathway (mobilizing intracellular Ca^++^) [31,35] and its activation promoted neural development/cell survival via the AKT (protein kinase B) pathway as well as a MAPK1/MAPK3 dependent cell proliferation [35]. Moreover, GAL 2 receptor stimulates MAPK pathways by the activation of protein kinase C and activates small GTPase proteins in the Rho family [35]. The stimulation of phospholipases C is also mediated by the GAL 2 receptor [35]. GAL 3 receptor is coupled to a Gα_i/o_ signalling pathway (involved in inward K^+^ currents) [31,35] and its activation promoted the inhibition of cAMP and adenylyl cyclase, altering the cAMP response element-binding protein (CREB) phosphorylation [35]. GAL 1 and 2 receptors mediate signalling cascades leading to adipogenesis, apoptosis and cell proliferation inhibition [35] by AKT/MAPK pathways. It has been reported that the 5-hydroxytryptamine (HT)_1A_-GAL 1/2 receptor heteromer (a macromolecular complex formed by at least for two different receptor units) is involved in the antidepressive action induced by GAL, since the interaction between these receptors induced conformational changes in GAL recognition sites altering GAL binding affinity [36]. Conformational changes reported in the GAL 1/GAL 2 receptor complex lead to a higher affinity of the GAL 1 receptor for the GAL_1-15_ fragment than for GAL, increasing the Gi/o mediated signalling which decreased both the level of CREB and the activity of adenylyl cyclase [37]. These mechanisms promoted depression. Thus, GAL promotes many signalling cascades depending on the ligand and G-protein type. However, to date, the native receptors for alarin, GAL-like peptide and GAL message-associated peptide have not been discovered [26].

As for feeding control mechanisms, GAL is mainly involved in fat consumption. Animals overexpressing GAL and GAL knockout, or wild-type animals showed a similar food intake when they received a standard diet [7]. Animals with a deletion of the GAL gene consumed less fat than control animals and the overexpression of GAL promoted a higher fat consumption [38]. After administration of a rich fat diet (45% fat), food intake/body weight increased in wild-type animals when compared to GAL knockout ones [7]. The administration of GAL had no effect on the preference for fat over carbohydrates/proteins, but the feeding response mediated by GAL was more prolonged and stronger in animals receiving a high-fat diet [38] (Table 1); when this diet was removed, GAL enhanced carbohydrate intake [2]. The administration of GAL into the hypothalamus promoted food intake in experimental animals [38]. GAL exerted the strongest action when was injected into the medial parvocellular division of the PVH and food intake progressively diminished when the injection of GAL moved from the lateral PVH to beyond (in all directions) the PVH [38]. It is known that the level of hypothalamic GAL increased in those animals showing a preference for fat [38] (Table 1). 

**Figure 3:**
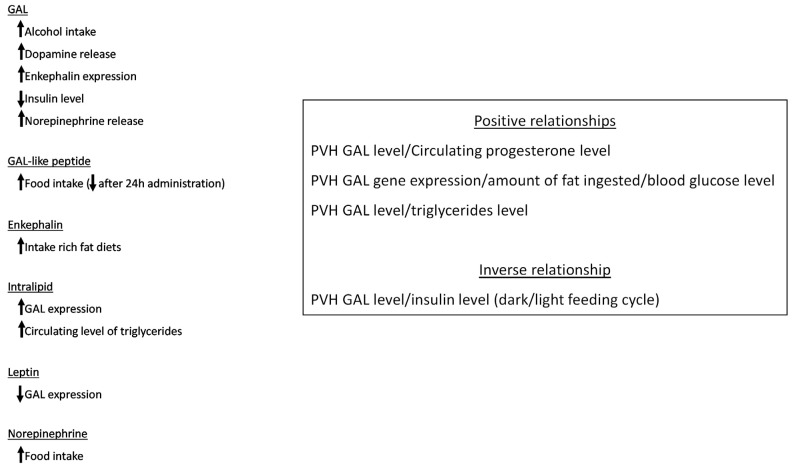
Paraventricular hypothalamic nucleus: physiological effects promoted by different substances when they were administered into the nucleus (left side). Some positive/inverse relationships are also shown (right side). ↑: increase; ↓: decrease. GAL: galanin; PVH: paraventricular hypothalamic nucleus.

Fat intake promoted an increase in both GAL level/expression in the middle/anterior parvocellular areas of the PVH, and the level of GAL was correlated with the amount of consumed fat [38] (Table 1; Figure 2). This means that the peptide is involved in a positive feedback loop which could facilitate an excessive fat intake [38] and that this feedback plays an important role in obesity (Table 1; Figure 2). In this sense, it seems that GAL, when food rich in fat is available, is involved in overeating/large meal size [38]. It is important to note that in the PVH, the GAL gene expression/synthesis of GAL was stimulated when a fat diet was administered but not when a protein or carbohydrate diet was consumed [6] (Figure 2). In this hypothalamic nucleus, the activation of the GAL 1 receptor increased adiposity, food intake and body weight (Table 1; Figure 2); however, according to the phenotypic analysis performed in mutant rodents deficient in GAL 2 receptors and the administration of GAL receptor-selective ligands, it seems that GAL 2 and 3 receptors are unlikely involved in the appetite stimulatory actions mediated by GAL [2,39,40,41,42,43]. Data suggesting that the GAL 1 receptor is involved in fat intake (Table 1) reported that the administration of the GAL 1 receptor agonist M-617 (Figure 2) promoted the intake of high-fat milk. In animals fed with a high-fat diet, control animals consumed more calories than those animals without the GAL 1 receptor gene [38], and a decrease in food intake was reported in GAL 1 receptor knockout animals [2]. GAL antagonists (e.g., M-40, galantide), administered into the PVH, decreased fat intake and this decrease was also observed when the level of GAL was reduced [38] (Table 1; Figure 2). Moreover, the administration of GAL into the PVH promoted alcohol intake (Figure 3) and the involvement of GAL in saccharin/sucrose intake has also been reported [38]. After food deprivation and when the stimulus for fat intake is high, the peptide/gene expression of GAL increased in the PVH (anterior parvocellular area); in addition, in free-feeding animals the level of GAL was high when the middle of the feeding cycle was reached (that is, when the preference for fat is increased) [38] (Table 1; Figure 2). After blocking fat oxidation, the expression of GAL decreased and that of the GAL 1 receptor increased in the PVH [38] (Figure 2). Finally, it has been suggested that fat promotes the proliferation/differentiation of developing nerve cells (located in the PVH), which finally express GAL [38]. 

GAL also exerts orexigenic effects in non-mammalian species. In chickens, GAL increased food intake in genetically selected low and high body weight lines and this effect was mediated through the Arc nucleus [44]. GAL is also involved in feeding in hens [35]; in this sense, the gene expression of GAL significantly increased in the jejunum of hens exposed to feed restriction [45]. In other peripheral tissues such are muscle cells, GAL stimulates carbohydrate metabolism over fat metabolism; by this way, GAL counteracts the metabolic disturbances due to a rich fat diet and in muscle cells, the peptide induces lipid partitioning away from oxidation toward storage in adipocytes [46]. Moreover, GAL decreased thermogenesis/energy expenditure by decreasing the activity of the sympathetic nervous system (which innervates the brown adipose tissue) and the expression of the uncoupling protein-1 that facilitates thermogenesis [47].

It is known that GAL-like peptide alters body weight/food intake in several species and that it is involved in energy metabolism [48,49,50]. GAL-like peptide is able to exert an orexigenic (inducing obesity) or an anorexigenic action [51] (Table 1). The former action is mediated by dopamine, orexin and NPY in the hypothalamus, whereas the latter is mediated by interleukins-1α and 1β, via the interleukin-1 receptor (interleukin-1 receptor antagonists attenuate the anorexigenic action of the GAL-like peptide) [52] (Table 1). Thus, this peptide exerts an anti-obesity action and accelerates lipid metabolism [48]. The GAL-like peptide is synthesized in mammalian hypothalamic neurons located in the Arc nucleus which also express leptin receptors [48]; these nerve cells send projections to several hypothalamic nuclei (e.g., PVH), targeting neurons that contain melanin-concentrating hormone or orexin [26], whereas neurons expressing GAL-like peptide receive inputs from projections containing NPY or orexin [26]. Finally, the involvement of GAL-like peptide in food intake has been demonstrated in other vertebrates such are goldfish, where the intracerebroventricular administration of the peptide increased food intake [53].

## 3. Galanin and Food Intake in Experimental Animal Models

Experimental animal models, mainly rodents, have been developed to study the mechanisms underlying feeding behaviour: from voluntary access, natural preference/selective breeding, restricted/intermittent access or forced exposure, to the molecular and cellular basis that modulate these behaviors. These models include animals with various induced pathologies up to transgenic animals, with overexpression or lack of a certain gene [6].

### 3.1. Rat

Obesity/overweight can promote metabolic complications such are hyperlipidemia or type 2 diabetes mellitus. A decrease in GAL plasma level has been reported in rat models with diabetes (type 2) compared with control animals [2] (Table 2). It has been demonstrated that the intracerebroventricular administration of GAL in animals with type 2 diabetes increased body weight, food intake, plasma adiponectin level, homeostasis model assessment-insulin resistance index, glucose transporter 4 mRNA expression and the concentration of this transporter in the plasma membrane and shows beneficial effects on insulin sensitivity [54] (Table 2). GAL blocks the release of insulin from pancreatic cells and facilitates glucose transporter 4 translocation onto plasma membranes [2] (Table 2). The administration of the GAL antagonist M-35 to control and diabetic rats decreased insulin sensitivity and the level of glucose transporter 4 (which mediates the transport of glucose into muscle cells and adipocytes) in the plasma membrane of both latter cells. The blockage of insulin release by GAL increases insulin sensitivity and glucose intolerance and accelerates the transport of glucose into cells [2] (Table 2). These effects, mediated via the GAL 2 receptor, were inhibited by the GAL 2 receptor antagonist M-871 and were similar (except for body weight and food intake) when the GAL 2 receptor agonist M-1,145 was administered [54]. The administration of a GAL 2 receptor agonist or antagonist does not alter body weight/food intake, and this means that the GAL 1 receptor (not the GAL 2 receptor) mediates high intake behaviours [54]. Thus, central GAL 2 receptor agonists exert a beneficial action on insulin sensitivity and hence these agonists could be used as an antidiabetic agent for the treatment of insulin resistance and diabetes (type 2). 

In a rat model of hyperphagia an excessive body weight gain was detected, and it was demonstrated that these animals were hyperresponsive to appetite-stimulated actions induced by GAL [55] (Table 2). During hyperphagia, GAL is upregulated in the Arc and parvocellular region of the PVH nuclei due to an increase in the levels of GAL and GAL gene expression in both nuclei [55] (Table 2; Figure 2). In genetically obese rats the hypothalamic GAL expression was enhanced [56] (Table 2) and it is known that GAL, when food is freely available, stimulated food intake in sated rats but this stimulation did not occur when an operant response requirement was present [57]. Thus, GAL shows dissociate effects when the food is freely available (increases consumption) and when an effort is required to obtain the food (motivation), in a way that even the most minimal contingence suppresses the GAL-induced increase of food consumption. This mechanism of decreasing motivation at times of high appetitive behavior is mediated by the GAL 1 receptor but not through the GAL 2 receptor [57] (Table 2).

Other brain regions are implicated in feeding behaviour and, in this sense, it is known that the hippocampus plays an important role in the modulation of food intake and hence in obesity [58,59,60,61,62]. A high-fat diet promoted cognitive deficits, hippocampal damage, an increase in serum cholesterol, and altered gene expression in different regions of the hippocampus before a significant fat mass/body weight increase [58]. Thus, in the rat dorsal hippocampus an acute high fat diet augmented the transcript levels of GAL, and in the ventral hippocampus a decrease in the level of the GAL 1 receptor has been observed [58] (Table 2). It is known that the actions mediated by GAL (e.g., the peptide could exert an opposite effect) are different depending on the CNS regions in which the peptide acts. The functional significance of these changes in the GAL/GAL receptor level must be determined; moreover, it is also important to know how females respond to an acute high fat diet since it seems that sex differences occur in the experimental model used [58].

The action of several compounds that induce changes in food intake has also been verified in animal models. It has been reported that the administration of daidzein reduced food intake in rats and that this anorexic effect was promoted by blocking the expression of GAL and by augmenting the CRH expression in the hypothalamus [63] (Table 2). In the control group, GAL mRNA levels were higher after feeding, whereas in the group treated with daidzein an increased level of CCK mRNA was observed in the small intestine [63]. Moreover, the hypothalamic expression of cocaine- and amphetamine-regulated transcript peptide (CART) and POMC and the levels of serum glucose, insulin and leptin were not affected by daidzein [63]. Thus, daidzein induces anorexia by controlling selectively the hypothalamic expression of GAL and CRH.

Regarding the action of other members of the GAL family on food intake, it is known that the administration of GAL-like peptide (e.g., into the rat PVH) increased the intake, but 24 h after administration both body weight and food intake decreased [26] (Table 2; Figure 3).

The GAL-like peptide also facilitates thermogenesis in rats. After the intracerebroventricular injection of the peptide, thermogenesis is mediated by the release of prostaglandin E2 from astrocytes of the periventricular hypothalamic nucleus, without the participation of neurons, or microglia, or peripheral tissues since the intravenous injection of the peptide did not produce thermogenesis. It has been suggested that these thermogenic effects, opposite to those induced by GAL, are due to an increase in heat production instead of a decrease in heat loss [64]. The intracerebroventricular administration of GAL-like peptide in rats increased significantly oxygen consumption (metabolic rate) and plasma levels of luteinizing hormone and decreased food intake [49], whereas the administration of GAL-like peptide into the nucleus of the solitary tract decreased significantly food intake and increased oxygen consumption and the plasma level of leptin, but not the luteinizing hormone level [49] (Table 2). Thus, GAL-like peptide decreases energy input acting at the level of the nucleus of the solitary tract, playing an important role in the hypothalamic control of the body weight, and increases energy expenditure [49] (Table 2). In diabetic rats, the expression of the GAL-like peptide was decreased when compared with control animals, but such expression returned to normal values when diabetic rats were treated with leptin or insulin [2] (Table 2). Finally, it has been reported that GAL-like peptide inhibits type 2 diabetes development and induces insulin resistance [2], and that insulin and glucose circulating levels regulate the expression of the GAL-like peptide gene [2] (Table 2).

### 3.2. Mouse

**Figure 4:**
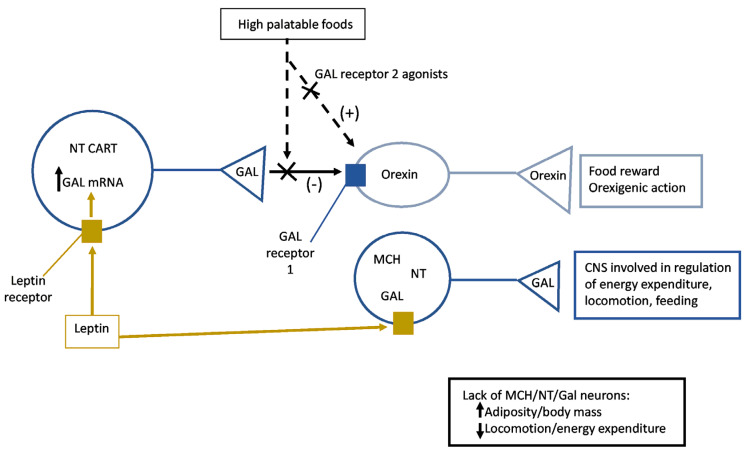
Extended perifornical area: neural pathway and neuroactive substances involved. Leptin inhibits the activity of neurons containing orexin. This inhibition is mediated by GAL after binding to the GAL receptor 1 expressed in neurons with orexin; however, high palatable foods block the inhibitory action of leptin on orexin neurons and stimulate the activity of the latter neurons. ↑: increase; +: activation (orexigenic action); -: inhibition (anorexigenic action). GAL: galanin; GAL receptor 2 agonists restore the ability of leptin to restrain palatable food eating. CART: cocaine- and amphetamine-regulated transcript peptide; MCH: melanin-concentrating hormone; NT: neurotensin.

Some of the neurons expressing GAL located in the extended perifornical area (perifornical area and adjacent lateral/dorsomedial hypothalamus) and in the nucleus of the solitary tract of the mouse brain also display leptin receptors [65]. The stimulation by leptin of these cells promoted the expression of GAL mRNA; this finding is not in agreement with the expected anorexigenic effect mediated by leptin [65] (Figure 4).

However, it is important to note that neurons co-expressing leptin receptors and GAL also express the anorexigenic CART and neurotensin peptides and are different from those nerve cells containing melanin-concentrating hormone or orexin [65] (Figure 4). It has been suggested that nerve cells co-expressing GAL and leptin receptors located in the extended perifornical area exert an inhibitory (anorexigenic) action instead of an orexigenic effect. It seems that the binding of leptin to its receptor located in nerve cells containing GAL promotes the release of GAL, which exerts an anorexigenic action by inhibiting the activity of neurons containing orexin. This has been observed in a mouse experimental model in which animals had a selective deletion of the leptin receptor from cells expressing GAL. This study focused on the involvement of the lateral hypothalamic neurons co-expressing the leptin receptor and GAL in the control of nutrient reward, showed that leptin regulated food reward mechanisms via the inhibitory action of GAL on nerve cells containing orexin [66] (Figure 4). Thus, GAL is a crucial mediator of the leptin action to regulate nutrient reward by inhibiting orexin neurons [66] (Table 2). In the mouse lateral hypothalamus, it has been reported that appetite-stimulating orexin neurons are activated when lean/satiated animals received a highly palatable food, and that this activation occurred even after the administration of leptin [67] (Figure 4). However, the administration of a GAL 2 receptor agonist into the lateral hypothalamus restored the ability of leptin to restrain palatable food eating, and this means that the inhibitory action promoted by leptin is mediated by the GAL 2 receptor [67] (Figure 4). It seems that the initiation of highly palatable food overeating occurs when the GAL inhibitory action on neurons containing orexin is blocked. Thus, during hedonic feeding, disinhibition of neurons containing orexin occurs. It is important to note that GAL neurons, but not nerve cells containing orexin, express leptin receptors. Thus, hedonically-loaded foods block the inhibitory actions of GAL on orexin neurons involved in hedonic overeating and, in lean mice, the overeating of palatable foods is decreased by activating the GAL 2 receptor in the lateral hypothalamus (Table 2). It is known that lateral hypothalamic neurons that regulate adiposity and energy balance and express the melanocortin-3 receptor also contain lateral hypothalamic markers (e.g., GAL, neurotensin, leptin receptor), and send projections to CNS regions involved in the regulation of energy expenditure, locomotion and feeding [68] (Figure 4). Food intake was not altered when the ablation of lateral hypothalamic neurons expressing the melanocortin-3 receptor was performed, whereas the lack of these neurons decreased locomotor activity/energy expenditure and increased adiposity/body mass [68] (Figure 4). The involvement of the different subpopulations of hypothalamic neurons expressing the melanocortin-3 receptor (e.g., also containing GAL or other neuroactive substances) in the previous mechanisms must be established since for example it is known that lateral hypothalamic neurons containing GAL regulate the locomotor activity [26]. 

In mice, some of the cells expressing GAL also express the neurosecretory protein GL. This protein promotes feeding behaviour and induces fat accumulation, and hence its dysregulation can induce metabolic alterations and obesity [69] (Table 2). In this sense, it has been demonstrated that protein GL regulates fat deposition via changes in locomotor activity and energy intake [69] (Table 2). Other studies have been focused on the mechanisms involving the comorbidity between abnormal eating behaviours and mood disorders [70]. In the human hypothalamus, spexin (a ligand for GAL 2/3 receptors) has been located in the PVH and supraoptic nuclei which are involved in many physiological actions (e.g., food intake, energy expenditure, osmotic homeostasis, stress) [71]. In experimental animal models, spexin exerted anorectic, antidepressive, anxiolytic and analgesic effects [72]. In underweight mice with signs of anxiety, depression, and anhedonia and with weight loss, it has been reported that a specific spexin-based GAL 2 receptor agonist administered into the lateral ventricle normalized body weight and mood behaviours [70]. In control mice, the agonist promoted weight loss probably by acting on hypothalamic CRH/POMC neurons (involved in body weight/appetite control) and on dorsal raphe serotonergic neurons (involved in mood control) [70] (Table 2). In addition, the intranasal administration of this agonist decreased, in a dose-dependent manner, body weight and food intake [70]. The data suggest the potential clinical use of spexin-based GAL 2 receptor agonists for the treatment of patients suffering from both abnormal body/appetite weight and mood disorders.

In the mouse, it has been reported that the administration of green tea extract (300 or 600 mg/kg/day for 51 days) decreased food intake, body fat/weight, prevented fat accumulation and increased the peripheral, but not the central, activity and expression of the endopeptidase neprilysin [73] (Table 2). This induced downregulation of orexigens (e.g., GAL, NPY) which are the substrates of neprilysin (Table 2) and, in fact, in neprilysin-knockout mice the green tea extract failed to decrease body weight/fat [73]. In sum, green tea extract decreased the level of body fat through the upregulation of neprilysin.

**Figure 5:**
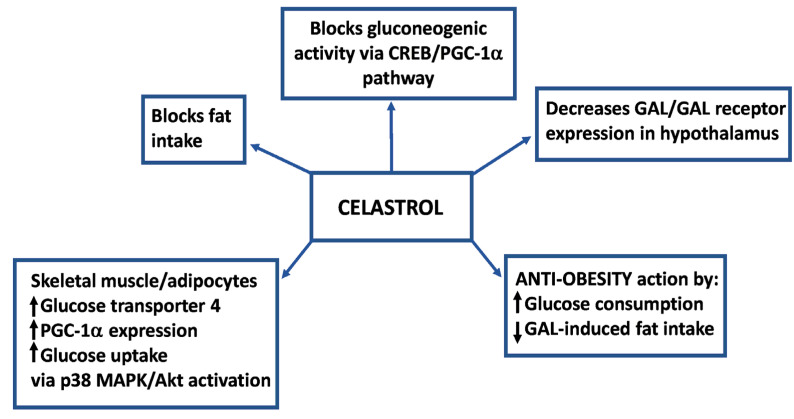
Effects of celastrol. ↑: increase; ↓: decrease. CREB: cAMP response element-binding protein; GAL: galanin; MAPK: mitogen-activated protein kinase; PGC-1α: peroxisome proliferator-activated receptor γ co-activator.

In another study, diet-induced obese mice were treated with the pentacyclic triterpene celastrol (100 μg/kg/day for 21 days) [74] (Table 2; Figure 5).

This compound, which crosses the brain–blood barrier, exerted an anti-obesity action because of decreased food intake and body weight. Celastrol blocked fat intake and induced weight loss by suppressing the expression of GAL and GAL receptors (GAL 1 and 3) in the mouse hypothalamus when these animals received a high-fat diet [74] (Table 2). Celastrol also decreased the expression of NPY/leptin in the hypothalamus and reduced GAL and GAL-like peptide concentrations in blood [48]. Moreover, celastrol blocked the gluconeogenic activity via a CREB/peroxisome proliferator-activated receptor γ co-activator (PGC)-1α pathway and augmented the glucose transporter 4 and PGC-1α expression to increase glucose uptake in skeletal muscle and adipocytes via p38 MAPK and AKT activation [74] (Figure 5). Thus, celastrol protects against obesity by activating the PGC-1α/glucose transporter 4 axis-mediated glucose consumption, and by suppressing GAL-induced fat intake [74]. This study demonstrates the anti-obese action mediated by celastrol and the potential use of this compound in clinical practice for the treatment of obesity (Figure 5). In addition, celastrol exerts an antidiabetic action because promotes an antihyperglycemic effect, reverts high fat diet-induced glucose intolerance and improves insulin resistance [74]. Thus, celastrol could be used in clinical practice to treat insulin resistance because increases insulin sensitivity and glucose uptake [74].

The relationships between GAL and glucose and insulin levels as well as triglycerides and cholesterol and the role of the diet have also been studied in experimental mouse models. In GAL transgenic male mice (GAL is overexpressed), the serum level of GAL was higher than that observed in control animals and, in addition, an increase in visceral adiposity, body weight and in the serum levels of triglycerides and cholesterol and a decrease in energy expenditure were reported [2] (Table 2). GAL or GAL 1 receptor gene knockout mice showed impaired glucose disposal due to a decrease in insulin response [2]. GAL-overexpressing or GAL-knockout mice did not show a lean or obese phenotype or any alteration in feeding behaviour respectively [26]. However, under macronutrient choice conditions and when fat is only available, GAL-knockout animals consumed less fat than control mice whereas GAL-overexpressing animals consumed more fat than control animals [26] (Table 2). The latter gained more bodyweight than GAL-knockout animals when a high-fat diet was administered [2] (Table 2).

As for other members of the GAL family of peptides, it has been reported that GAL-like peptide knockout animals, compared to control ones, gained less weight after the administration of a rich fat diet [2] (Table 2). Compared to the control group, mice with diet-induced obesity (high-fat diet for two weeks) and following an intranasal administration of GAL-like peptide showed a lipid accumulation inhibition in the liver and a decrease in body weight [75]. In treated animals, the levels of hepatic triglycerides and lipid droplets in hepatocytes decreased, but the hepatic fatty acid β-oxidation-related gene mRNA level was increased [75]. In leptin-deficient obese mice, the chronic administration of GAL-like peptide promoted a decrease in body weight compared to control animals [26]. In obese mice, the uptake of the GAL-like peptide by the brain was higher after an intranasal administration of the peptide than after an intravenous administration [76]. The peptide decreased food intake and body weight gain (at a dose of 1-2 nmol, but not at 4 nmol) and the intranasal administration of GAL-like peptide promoted the hypothalamic expression of c-fos in the lateral, dorsomedial and Arc hypothalamic nuclei [76]. It seems that GAL-like peptide decreases food intake via interleukin-1 signalling pathways and no effect on food intake and body weight gain was observed when young lean mice received an intranasal administration of GAL-like peptide [76]. The results indicate that the intranasal administration of GAL-like peptide favours energy expenditure and blocks energy intake and that the peptide decreases body weight in obese mice showing a high level of glucose in blood [76]. Thus, the intranasal administration is an effective route by which the GAL-like peptide can exert an anti-obesity action (Table 2). In spontaneously exercising mice, it has been reported that the intracerebroventricular administration of GAL-like peptide favoured energy metabolism (Table 2); the peptide promoted both lipid and glucose metabolisms and inhibited the synthesis of fatty acids and gluconeogenesis [50]. These animals increased heat production level/consumed oxygen volume and decreased body weight (Table 2) and sterol regulatory element-binding protein-1c (which regulates fatty acid synthesis), phosphoenolpyruvate carboxykinase (which regulates gluconeogenesis) and glucose transporter-4 mRNA expressions [50]. The data suggest that the reduction in body weight and the decrease in phosphoenolpyruvate carboxykinase mRNA expression are related to the interleukin-1α/1β-mediated action of the GAL-like peptide [50]. According to the results obtained, this peptide can be considered as an anti-obesity candidate, but more studies (e.g., administration mode, dose optimization) are necessary to confirm this action. It is important to note that the blood–brain barrier controls the passage of peptides involved in food intake from blood to brain and this control occurs in several physiological states [77]. Thus, fasting or hyperglycemia can respectively downregulate or upregulate the transport of GAL-like peptide through the blood–brain barrier [77] (Table 2). Finally, it seems that under altered dietary situations, GAL-like peptide is involved in the maintenance of metabolic homeostasis although its involvement in energy balance mechanisms is not pivotal [26] (Table 2).

It is known that the hypothalamic dorsomedial nucleus is involved in the food intake mediated by alarin [78]. Alarin exerts an orexigenic action since increases body weight, and the peptide also increases the plasma level of luteinizing hormone [78]. In the mouse, alarin 6-25Cys (a peptide fragment missing five residues at the amino-terminal end) antagonizes both previous effects and hence the elimination of the first five amino acids of the full-length alarin abolishes the biological effects of alarin [78]. In male mice, the intracerebroventricular administration of alarin increased food intake, body weight and the level of luteinizing hormone (Table 2) and stimulated the expression of c-fos in diencephalic nuclei (e.g., dorsomedial hypothalamic nucleus), but exerted no effect on body temperature [32]. These results agree with those reported in male rats in which alarin exerted an orexigenic effect and regulated the secretion of reproductive hormones [32]. Moreover, it seems that alarin is not involved in the metabolic rate central regulation [32].

### 3.3. Pig

In this animal model, studies have been carried out on the influence of certain substances present in food or in food containers on the enteric system and especially on the expression of diverse neuropeptides, among them, GAL. Acrylamide, which exerts toxic effects in rodents and humans, is contained in food (e.g., coffee, fries, chips) and absorbed mainly in the small intestine which is directly exposed to its toxic effect [79]. Daily administration of low (0.5 μg/kg) and high (5 μg/kg) doses of acrylamide to pigs for four weeks produced changes in the neurochemical characteristics of the neurons located in the duodenum and belonging to the enteric nervous system [79]. In comparison to control animals, both low and high doses of acrylamide increased the number of immunoreactive neurons containing GAL, substance P or calcitonin gene-related peptide [79] (Table 2). The number of the immunoreactive neurons increased when the dose of acrylamide also increased [79]. The authors of the study concluded that the above three mentioned peptides could protect the enteric nervous system neurons against the harmful effect mediated by acrylamide, since it is known, for example, that GAL is involved in neurotrophic mechanisms [79] (Table 2). However, it is also known that GAL, substance P and calcitonin gene-related peptides play important roles in inflammatory mechanisms. In animals treated with acrylamide, the higher expression of the three neuropeptides could be also related to the inflammatory processes observed after its administration [79]. Moreover, in the pig, the action of low or high doses of acrylamide on GAL-immunoreactive neurons located in the stomach was studied [80]. In both submucous and myenteric plexuses, an upregulation of the GAL-immunoreactivity was observed, and the proportion of neurons co-expressing GAL and vasoactive intestinal peptide or nitric oxide synthase was also augmented [80]. Like the previous study [79], the authors suggest that GAL could play a neuroprotective and/or neurotrophic action involved in the recovery of the stomach enteric nervous system neurons after an intoxication mediated by acrylamide (Table 2). However, the involvement of GAL in the inflammatory mechanisms promoted by acrylamide cannot be discarded.

The administration of bisphenol A (an organic compound found in plastics used as water/food containers) induces changes in the enteric nervous system of the pig duodenum [81]. The neurochemical characterization (e.g., expression of GAL, substance P, vasoactive intestinal polypeptide, CART) of the enteric neurons was studied after a 7-day administration of low (0.05 mg/kg/day) and high (0.5 mg/kg/day) doses of bisphenol A [81]. The results showed important changes in duodenal immunoreactivity for the above-mentioned neuropeptides even at a low dose, and this means that bisphenol A affects the neurochemical characterization of neurons belonging to the enteric nervous system, displaying a severity related to the dose of bisphenol A administered [81] (Table 2). After the administration of bisphenol A (0.05 mg/kg/day (low dose) or 0.5 mg/kg/day (high dose) for 28 days), changes have also been observed in the intrahepatic sympathetic fibers of the pig regarding the expression of GAL, substance P, calcitonin gene-related peptide and CART [82]. Low and high doses of bisphenol A increased the number of intrahepatic sympathetic fibers and the number of these fibers containing GAL [82] (Table 2). This means that bisphenol A alters the sympathetic innervation of the liver which may induce changes in the cellular metabolism/oxygenated blood supply affecting the function of hepatocytes.

## 4. Galanin and Food Intake in Humans

Thin men and women display a lower plasma level of GAL than individuals with obesity [33,83], and the level of GAL in the serum is high in obese menopausal women, but this level is decreased in the cerebrospinal fluid of recovered anorexic women [38] (Table 3). In women with moderate/severe obesity a high plasma concentration of GAL has been reported [56,84] (Table 3). A study focused on GAL alleles/body weight did not report differences between healthy and obese young adults [85]. This suggests that the augmented plasma level of GAL was due to obesity and not to food intake, and hence obesity increases the secretion of GAL [84] (Table 3). 

In healthy subjects and in those suffering from type 2 diabetes, a positive relationship between blood glucose and GAL levels has been reported [2] (Table 3). Impaired glucose tolerance is a prediabetic stage since it appears before the development of type 2 diabetes and a high level of plasma GAL is related to the development of impaired glucose tolerance [86]. Moreover, in patients with impaired glucose tolerance, a high level of GAL has been associated with hyperglycemia [86]. Serum levels of GAL, glucose and insulin have been measured in patients suffering from impaired glucose tolerance and in control subjects with normal glucose tolerance [86]. One-two hours after food intake, the levels of GAL, glucose and insulin were higher in the former group than in controls and, in addition, body weight was higher in patients with impaired glucose tolerance [86]. In these patients, a negative correlation between one-hour glucose concentration and GAL level was found [86] (Table 3). These results suggest that in patients with impaired glucose tolerance an interaction between GAL and insulin occurs, thus they can promote a mutual secretion/synthesis inhibition, and that the enhanced GAL serum level could be used as a biomarker for the prediction of impaired glucose tolerance (Table 3). To confirm the results reported, a larger population of patients must be studied. Non-pregnant women suffering from type 2 diabetes showed a high level of circulating GAL and this level was positively correlated with the level of blood glucose [83] (Table 3). The circulating GAL level was also higher in pregnant women with gestational diabetes mellitus than in pregnant women with normal glucose tolerance [83]. Moreover, a positive correlation was observed between the levels of glucose and GAL in pregnant women with gestational diabetes mellitus [83] (Table 3). Thus, it seems that GAL upregulation was related to the high level of glucose, that the circulating GAL level, observed in the latter women, is associated with changes in blood glucose and that the higher level of GAL observed in pregnant women with gestational diabetes mellitus is a physiological adaptation due to the rise of glucose associated to diabetes [83], although a pathophysiological adaptation must not be discarded. Moreover, the level of GAL was not correlated with that of insulin and it seems that this could be due to insulin resistance mechanisms [83].

In healthy and obese children, the relationships between GAL serum levels and metabolic parameters (insulin, glucose, lipids, homeostasis model assessment-insulin resistance, leptin) have been studied [87]. Compared to healthy individuals, serum levels of both leptin and GAL were higher in obese children and the levels of GAL were positively correlated with the levels of fasting insulin, homeostasis model assessment-insulin resistance, triglycerides, and fasting glucose [87] (Table 3). Thus, in obese children, serum levels of GAL are positively correlated with triglycerides and insulin resistance. The results indicate that GAL is associated with lipid metabolism and glucose homeostasis in these children [87] (Table 3) and suggest that GAL is involved in the development of obesity and associated metabolic disorders. The increase in GAL could be due to the appearance of GAL tissue resistance or might be a compensatory mechanism against the insulin sensitivity increase observed in obese subjects suffering from insulin resistance [87]. In obese children/adolescents, no allelic difference in GAL 1 receptor or GAL has been reported when compared to healthy subjects and no association has been found between GAL 2 receptor and obese phenotypes [38] (Table 3). The rs2187331 allele has been related to a high level of circulating triglycerides [38]. In obese (non-diabetic) or healthy lean young men the serum level of GAL was measured during an oral glucose tolerance test [71]. The study showed that the GAL level was lower in lean subjects than in obese individuals and that the serum GAL level was positively correlated with the levels of leptin, triglycerides, total cholesterol and fat, body mass index and visceral fat and negatively correlated with adiponectin [71]. Moreover, it was suggested that the serum insulin level is a predictor of the serum level of GAL and that the high serum level of GAL observed could be associated with the increase in body weight reported in obese (non-diabetic) individuals and to insulin resistance [71]. The results also suggest the occurrence of GAL resistance in obese subjects.

Another member of the GAL family, alarin, increases food intake and body weight [88,89]. An immunocytochemical study on the distribution of alarin in the human intestinal epithelia described that alarin is present in Paneth cells (also expressing α-defensin 5) and in entero-endocrine cells (also expressing chromogranin and synaptophysin) in the small bowel [88]. In the large bowel, the co-existence of alarin and α-defensin 5 was not visualized, but co-existence was observed in cells containing alarin, chromogranin, synaptophysin and somatostatin; in cells co-expressing alarin, 5-HT and chromogranin, or in cells co-expressing alarin, 5-HT and PYY [88]. The functional roles (e.g., food intake, gut motility, hormone release regulation) played by the above-reported substances located in human entero-endocrine and Paneth cells must be elucidated in future studies. In experimental animals, alarin improved insulin resistance since decreased the levels of insulin and blood glucose [89]. Moreover, in humans, a high circulating alarin level has been associated with metabolic syndrome (abdominal central obesity, hypertension, glucose intolerance, atherogenic dyslipidemia) and insulin resistance [2,89] (Table 3). The level of alarin was higher in metabolic syndrome patients than in healthy subjects and, in addition, circulating alarin levels were positively correlated with blood pressure, fasting blood glucose, AUCglucose, glycated haemoglobin, homeostasis model assessment of insulin resistance), triglyceride, waist circumference and tumor necrosis factor α [89] (Table 3). In healthy subjects, acute hyperglycemia, lipid infusions and glucose challenge promoted an increase in circulating alarin levels, whereas acute hyperinsulinaemia transiently decreased these levels [89]. Thus, it seems that a high level of circulating alarin could be beneficial for patients suffering the metabolic syndrome and that the high level of alarin observed in these patients could be a compensatory mechanism against the metabolic stress promoted by hyperlipemia, obesity or hyperglycemia [89].

Spexin, a peptide involved in energy metabolism regulation, exerts an anti-obesity action since decreases body weight and blocks food intake, lipogenesis, and the uptake of glucose [90] (Table 3). In obese humans, spexin is the most downregulated gene in fat and it was reported that the actions exerted by spexin on the metabolism of adipocytes were mediated by GAL 2 and GAL 3 receptors and that the peptide could be considered as a regulator of the lipid metabolism [90] (Table 3). In human adipocytes, spexin and its GAL 2 and GAL 3 receptors are present at protein/mRNA levels and the peptide blocks adipogenesis and downregulates the mRNA expression of some pro-adipogenic genes [90] (Table 3). Spexin promotes lipolysis in human adipocytes and regarding the control of fat cell metabolism, it is important to note that the effects promoted by spexin are contrary to those exerted by GAL [90]. Both spexin and GAL are agonists of the GAL 2/3 receptors; however, GAL, but not spexin, activates the GAL 1 receptor and this could explain the opposite actions observed [90]. Thus, the anorexigenic peptide spexin could be used to treat obesity.

A recent review has been focused on single-nucleotide polymorphisms (SNPs, mutation/change in one DNA base pair in a gene) in orexigenic neuropeptides (e.g., GAL) targeting G-protein coupled receptors [7]. These SNPs can be involved in altered receptor affinity/signalling for peptides pathways related to appetite, as well as in altered functional activity/expression level of these peptides [7]. To date, in humans, no SNP has fully been associated with obesity or altered body mass index [7] (Table 3). It has been suggested that the C-terminal domain of GAL prevents the degradation of the peptide [7] and that the N-terminal domain 16 residues of GAL is pivotal for receptor–ligand function, of which 12 positions have an SNP in humans [7].

## 5. Relationships Between Galanin and Other Substances

**Figure 6:**
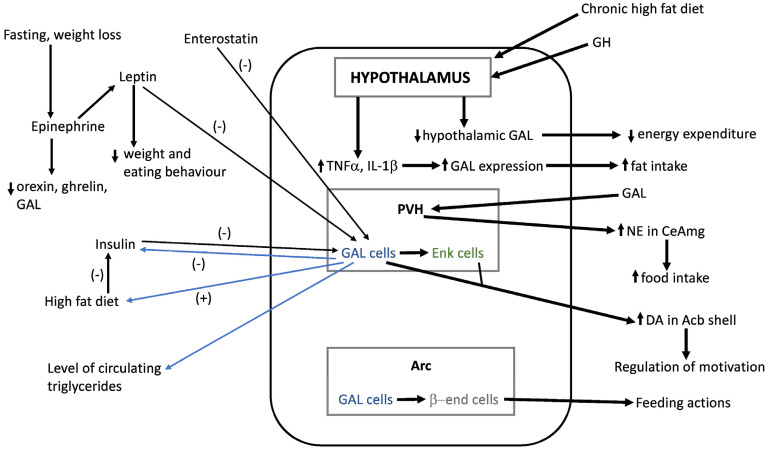
Molecular interactions between GAL and other substances. General interactions (i.e., hypothalamus) are indicated, and more specific actions are signaled separately. Actions mediated specifically by GAL are represented by blue arrows. ↑: increase; ↓: decrease; +: activation; -: inhibition. Acb: accumbens nucleus; b-end: beta-endorphin; CeAmg: central nucleus of the amygdala; DA: dopamine; Enk: enkephalin; GAL: galanin; GH: growth hormone; IL-1β: interleukin 1-beta; NE: norepinephrine; PVH: paraventricular hypothalamic nucleus; TNFα: tumor necrosis factor-alpha.

In this section, the relationships between GAL and dopamine, norepinephrine, epinephrine, enkephalin, beta-endorphin, CART, enterostatin, insulin, leptin, growth hormone, progesterone, cytokines, triglycerides and NPY will be mentioned (Figure 6). 

### 5.1. Dopamine

GAL promotes feeding intake by increasing the level of dopamine in the nucleus accumbens (Table 4) and it is also known that dopaminergic nerve cells located in the ventral tegmental area project to the nucleus accumbens and express GAL receptors. The administration of GAL into the ventral tegmental area facilitates the accumulation of dopamine metabolites into the nucleus accumbens [38]. The administration of GAL into the PVH increased the extracellular level of dopamine in the nucleus accumbens shell and regulates reward mechanisms (Figure 6), such as a meal rich in fat [38] (Figure 3). The data suggest that PVH neurons project to the nucleus accumbens shell. This diet increased the levels of GAL and dopamine respectively in the PVH and nucleus accumbens shell [38]. Thus, it seems that the release of dopamine-mediated by GAL into the nucleus accumbens shell is involved, not in the consumption of fat, but in the motivational processes regulating such consumption [38] (Table 4; Figure 6).

### 5.2. Norepinephrine

In satiated rodents, norepinephrine promoted food intake after its administration into the PVH (Table 4; Figure 3) where GAL and norepinephrine exerted the strongest food intake signal compared to other CNS nuclei [38]. In the PVH, GAL and norepinephrine coexist in nerve cells, in which the peptide facilitates the release of norepinephrine [38]. The administration of GAL into the PVH increased the level of norepinephrine (Figure 3; Figure 6), and substances that inhibit the synthesis of norepinephrine/alpha 2-noradrenergic receptor blocked the feeding intake promoted by GAL; thus, the feeding mechanisms mediated by the peptide were due to the effects promoted by the release of norepinephrine [38] (Table 4). Moreover, it has been reported that the feeding mechanisms induced by GAL were mediated by noradrenergic alpha-2/5-HT_1A_ receptors [2] (Table 4), and that the orexigenic effect mediated by GAL was blocked when the synthesis of norepinephrine was inhibited [91]. It seems that the orexigenic effect of GAL was also due to the activation of the GAL 1 receptor/5-HT_1A_ receptor heteromers which blocked the 5-HT_1A_ activation and hence an increase in food consumption occurred [91] (Table 4). In this sense, it is known that GAL exerted an inhibitory action on the physiological functions mediated by the 5-HT_1A_ receptor and that 5-HT_1A_ receptor agonists administered into the lateral hypothalamus decreased food consumption [91].

It is also known that GAL increases food intake when was injected outside the hypothalamus [38]. In the central nucleus of the amygdala, food intake is mediated by norepinephrine (Figure 6) and because this is a stress-related neurotransmitter, it seems that GAL mediates stress-induced overconsumption [26] (Table 4). 

### 5.3. Epinephrine 

Fasting and weight loss promote an increase in the level of epinephrine [92]. It has been reported that epinephrine blocked starvation-induced secretion of orexin, ghrelin and GAL in starved (50% energy requirement) and normal (100%) male rats, and that the response to epinephrine might be affected by the weight loss [92] (Table 4; Figure 6). It seems that leptin is also involved in these processes, since this hormone is the target of epinephrine [92]. 

### 5.4. Enkephalin

In the PVH, GAL interacts with enkephalin (Table 4; Figure 6) since the administration of the opioid peptide into this nucleus increased the consumption of rich fat diets (Figure 3) over rich carbohydrate diets. In addition, analogues of enkephalins increased the level of dopamine in the nucleus accumbens shell [38]. Fat ingestion promoted the expression of both enkephalin and GAL in nerve cells placed in the parvocellular area of the PVH [38] (Table 4; Figure 2). The hypothalamic administration of GAL increased the expression of enkephalin in the PVH (Figure 3), and it seems that this mechanism was due to the inhibition (via the GAL 1 receptor) of the nerve cells containing GABA located in the PVH [38] (Figure 2). Thus, fat intake is mediated by the GAL action on enkephalinergic neurons located in the PVH and, in fact, fat ingestion mediated by GAL was blocked with µ-opioid receptor antagonists (e.g., naloxone) [38] (Table 4; Figure 2).

### 5.5. Beta-Endorphin

It is known that beta-endorphin is involved in feeding [93], and that Arc boutons containing GAL from local origin cells form synaptic contacts with dendrites/cell bodies containing beta-endorphin [93]. The functional action of this close neuroanatomical relationship has been also reported, since food intake mediated by GAL is, in part, due to the release of beta-endorphin which is controlled by GAL [93] (Table 4; Figure 6). 

### 5.6. Cocaine- and Amphetamine-Regulated Transcript Peptide (CART)

CART regulates food intake in the CNS [94]. Using double- and triple-immunofluorescence techniques, the wide distribution of this peptide and its co-existence with other neuroactive substances has been reported in the enteric nervous system of the pig oesophagus [94] (Table 4). According to the co-existence pattern observed, CART was located in stimulatory or inhibitory neurons suggesting that the peptide exerts different physiological roles in the oesophagus. In addition, the physiological actions mediated by GAL must be determined and it seems that these actions depend on the intestine segment in which cells containing the peptide and CART are located [94].

### 5.7. Enterostatin

This peptide is synthesized according to the amount of fat ingested. Enterostatin decreases fat intake, specifically blocks the stimulated feeding action mediated by GAL (Figure 6) and does not affect the carbohydrate intake, a mechanism mediated by NPY [56] (Table 4).

### 5.8. Insulin

Insulin decreases gene expression of GAL in PVH (Figure 6). After a high-fat diet, the level of insulin decreased, and this could promote the overexpression of GAL in the PVH [56] (Table 4). A positive relationship has been reported between the amount of fat ingested, the gene expression of GAL in the PVH and the level of blood glucose [56] (Table 4; Figure 3). The administration of GAL into the PVH decreased the level of insulin (Table 4; Figure 3), but this was increased when an antisense oligonucleotide to GAL mRNA was administered into the PVH [56]. Moreover, along the dark/light feeding cycle, an inverse relationship has been reported between insulin and GAL levels in the PVH [34] (Table 4; Figure 3). It is known that the administration of insulin into the medial hypothalamus decreased the expression and release of GAL and NPY [95]. In diabetic rats (type 2 and receiving a high-fat diet) it has been demonstrated that, in comparison with insulin glargine, insulin detemir promoted less food intake and weight gain and that this was due to the downregulation of hypothalamic GAL mRNA and protein expressions [95] (Table 4).

### 5.9. Leptin

The hormone leptin, produced in the adipose tissue, decreases body weight, eating behaviour and GAL expression in the hypothalamus (e.g., PVH), regulating the ingestion of fat [56,96] (Table 4; Figure 3; Figure 6). It has been reported that GAL blocked the sensitivity of fat tissue/body weight to leptin and that the intravenous administration of GAL reduced in the visceral adipose tissue the synthesis/secretion of leptin which plays an important role in controlling energy balance/feeding [97] (Table 4).

### 5.10. Growth Hormone

The release of GAL facilitates the adaptation of metabolism mechanisms and fat intake in response to changes in the level of the growth hormone [2]. Thus, the administration into the hypothalamus of this hormone promoted a decrease in the level of hypothalamic GAL leading to a reduction in energy expenditure and lipid oxidation (Figure 6), although changes in both GAL hypothalamic level and food intake were gradually attenuated until reaching normal values [2] (Table 4).

### 5.11. Progesterone

During the proestrous period, a positive relationship has been observed between circulating progesterone and GAL levels in the median eminence, PVH and medial preoptic nucleus [56] (Table 4; Figure 3). It is important to note that the PVH is involved in fat intake in female rats but not in males, and it seems that progesterone increases the expression of GAL in the medial preoptic nucleus in females [56] (Table 4). Moreover, in the medial preoptic nucleus the level of GAL was positively correlated with body fat (adiposity) in female rats but not in males, and hence progesterone could play an important role in the development of obesity in females with a high-fat diet consumption [56] (Table 4). In female rats, fat intake increases the hypothalamic level of GAL as well as the circulating levels of progesterone and oestrogen, and this also occurs in ovariectomized animals after the administration of estradiol [38]. In control females during the proestrus phase, the level of GAL increased in the hypothalamus in which both progesterone/oestrogen levels augmented; in addition, the preference for fat intake also augmented [38]. In castrated males treated with testosterone or oestrogen the hypothalamic expression of GAL increased [38]. Moreover, the positive relationship between increased fat intake, GAL and sex steroids has also been observed in female rats during puberty [38], and it has been suggested that one of the signals for female puberty onset is mediated by steroids which stimulate GAL to increase fat intake [38] (Table 4).

### 5.12. Cytokines

In the hypothalamus, the chronic administration of a fat-rich diet increased the levels of tumor necrosis factor-alpha and interleukin 1-beta which stimulated the gene expression of GAL [38] (Table 4; Figure 6). It seems that the increase in the level of GAL counteracts the harmful effects mediated by inflammatory cytokines after a chronic intake of fat [38] (Table 4).

### 5.13. Triglycerides

In the PVH, the expression of GAL is positively related to the circulating level of triglycerides (increased levels, a higher GAL expression) (Figure 6) and it has been reported that GAL is related to saturated fatty acids intake [38] (Table 4; Figure 3). The administration of intralipid increases both the expression of GAL in the PVH and the level of circulating triglycerides [38] (Table 4; Figure 3). Fatty acids, arising from the hydrolysis of the circulating triglycerides, can cross the blood–brain barrier and exert a central action [38]. Currently, this action is unknown on the expression of GAL (Figure 2) although it is known that animals overexpressing GAL showed a higher level of circulating triglycerides [38] (Table 4).

### 5.14. Neuropeptide Y

**Figure 7:**
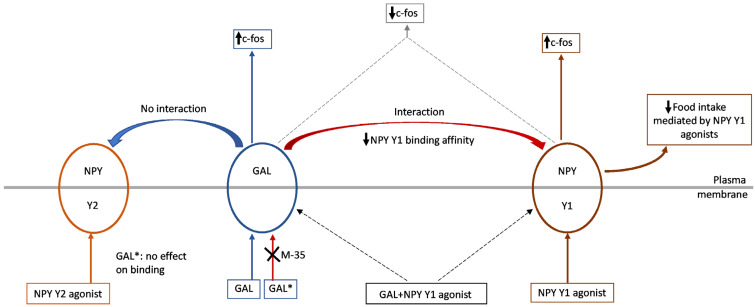
GAL receptor–NPY receptor interaction (arcuate nucleus): antagonistic GAL receptor modulation of NPY Y_1_ receptor mechanisms. GAL (threshold dose; no effect on food intake when administered alone) blocks food consumption mediated by NPY Y_1_ receptor agonists. GAL, after binding to its receptor, decreases NPY Y_1_ receptor agonist affinity and signalling; this was counteracted by the GAL receptor antagonist M-35. The administration of GAL (a dose that promotes food ingestion) or NPY Y_1_ receptor agonist increased the expression of c-fos, but the co-administration of both compounds decreased this expression. GAL^*^ (threshold dose); ↑: increase; ↓: decrease; X (inhibition). GAL: galanin; NPY: neuropeptide Y.

Because NPY is one of the main peptides most involved in the regulation of intake, the interactions between NPY and GAL have been extensively studied. In cell cultures, GAL promotes the secretion of NPY by hypothalamic neurons [38] and, in some areas of the CNS, a close neuroanatomical relationship between GAL and NPY occurs [98] (Table 4). Thus, nerve cells expressing NPY or GAL show synaptic contacts with each other in several hypothalamic nuclei (e.g., Arc, PVH) and functional GAL/NPY interactions have been reported in feeding behaviour and energy metabolism [98,99] (Table 4). In this sense, in the hypothalamus of NPY-knockout mice GAL mRNA level was upregulated, indicating that GAL can functionally compensate for the loss of NPY [99]. In rats, it has been reported that the threshold dose of GAL inhibited the food consumption mediated by the NPY Y_1_ receptor agonist [^125^I] Leu^31^, Pro^34^ PYY, while no effect was observed regarding food intake when administered alone into the lateral ventricle, and in addition, this inhibition was not observed when an NPY Y_2_ receptor agonist was administered [99] (Table 4; Figure 7). 

This finding suggests that a blockade of the NPY Y_1_ receptor-mediated effect occurred by means of a specific GAL receptor–NPY Y_1_ receptor interaction, since the administration (alone) of the above NPY Y_1_ receptor agonist increased food intake [98] (Figure 7). In fact, at the plasma membrane, the GAL receptor interacts with the NPY Y_1_ receptor in the rat Arc [98,99] (Table 4). Thus, in this nucleus, GAL decreased the binding of the NPY Y_1_ receptor agonist [^125^I] Leu^31^, Pro^34^ PYY; an effect that was counteracted by the specific GAL receptor antagonist M-35 [99] (Table 4; Figure 7). However, no GAL effect on [^125^I] PYY 3-36 (NPY Y_2_ receptor agonist) binding characteristics was reported [99]. In sum, in the rat Arc, GAL inhibits food intake mediated by NPY Y_1_ receptor agonists through a GAL receptor–NPY Y_1_ receptor interaction [98] (Table 4). Immunocytochemical studies performed to show the expression of c-fos as a neuronal marker confirmed that, in the Arc, GAL regulated the food intake promoted by the above-mentioned NPY Y_1_ receptor agonist [99]. The intracerebroventricular administration of this agonist or GAL alone increased c-fos expression in the Arc [98] (Figure 7). However, the co-administration of the above NPY Y_1_ receptor agonist and GAL (at a dose that stimulates food intake) decreased c-fos expression, promoted by the NPY Y_1_ receptor agonist, in Arc [99] (Figure 7). Taken together, these results show a specific antagonistic GAL receptor modulation of NPY Y_1_ receptor mechanisms but no effect on NPY Y_2_ receptor agonist binding, and suggest that an interaction between both receptors occurs leading to a decrease in NPY Y_1_ receptor agonist affinity which takes place in a receptor heteromer (GAL receptor/NPY Y_1_ receptor) [99] (Figure 7). In this way, GAL blocks food intake mechanisms mediated by NPY Y_1_ receptor agonists in the rat Arc: GAL can promote conformational changes in its receptor which can also induce conformational changes in the NPY Y_1_ receptor. These changes reduced NPY Y_1_ receptor recognition and G protein coupling and hence decreased the NPY Y_1_ receptor signalling [98]. Previous findings are in agreement with other observations reporting the presence of neuropeptide receptor heteromers (e.g., GAL 1 receptor/µ-opioid receptor) in certain regions of the CNS (e.g., ventral tegmental area) [100]. In the case of the hypothalamus-ventral tegmental area neuroanatomical pathway, heteromers have been suggested as targets for the treatment of food intake control loss [100].

It is also known that NPY neurons located in the Arc send inhibitory inputs to neurons containing GAL placed in the PVH [38] (Table 4; Figure 1; Figure 2). This means that carbohydrate intake, regulated by NPY, decreases fat intake due to the inhibition exerted by NPY on neurons containing GAL [38] (Table 4). In this sense, a high-carbohydrate diet increases the levels of NPY and decreases GAL levels in the PVH [38] and, in addition, rats consuming more fat than carbohydrates showed a higher expression of GAL than NPY in the same hypothalamic nucleus [38] (Figure 2). The interaction between GAL/NPY has also been described in other conditions such as maternal obesity by consumption of high-fat diets or postnatal overnutrition. In both cases, it has been reported a decrease in the expression of anorexigenic peptides as well as an increase in the number of neurons containing orexigenic peptides (NPY, GAL) in the PVH and lateral hypothalamus [5]. Thus, the study of peptidergic interactions should not be limited to adults.

## 6. Therapeutic Strategies

Currently, no effective pharmacological treatment for obesity has been developed, and in many cases, important side-effects appeared when anti-obesity treatments were applied. Some compounds have been tested in experimental animals to treat obesity: daidzein, spexin-based GAL 2 receptor agonists, spexin and celastrol [63,70,74]. Daidzein exerts an anorexic action by modulating the expression of GAL, NPY, CRH and CCK [63], whereas spexin-based GAL 2 receptor agonists normalized body weight and mood behaviour by modulating CRH, POMC and serotonergic neurons [70]. Comorbidity between abnormal eating behaviours and mood disorders is frequent, thus spexin-based GAL 2 receptor agonists are potential clinical compounds to be administered to subjects suffering from mood disorders and abnormal body/appetite weight. Spexin inhibited glucose uptake/lipogenesis and decreased body weight/food intake, exerting an anti-obesity action. In the case of celastrol, this pentacyclic triterpene exerts an anti-obesity action by suppressing GAL-induced fat intake and by activating the PGC-1α/glucose transporter 4 axis-mediated glucose consumption. Although these preclinical studies show an anti-obesity action and potential use of these compounds in clinical practice for the treatment of obesity, additional preclinical data must confirm the results currently reported. Thus, for example, it is important to know in-depth the molecular mechanisms by which celastrol targets the GAL/GAL receptor system and decreases food intake and body weight, as well as those mechanisms by which celastrol exerts an antidiabetic action.

Heteromers (receptor–receptor interaction) are potential targets for food intake dysregulation. In this sense, orexigenic mechanisms mediated by GAL are due to the activation of GAL 1 receptor/5-HT_1A_ receptor heteromer which blocks 5-HT_1A_ activation [91]. Thus, a promising research line to know the molecular mechanisms involved in feeding behaviour is to determine receptor–receptor interactions in hypothalamic nuclei involved in this behaviour as potential therapeutic targets. On the other hand, feeding mechanisms mediated by GAL are due to the effects promoted by the release of beta-endorphin or norepinephrine and this means that a food intake decrease could be possible by targeting both neuroactive substances.

## 7. Conclusions

Obesity/overweight are important health problems due to metabolic complications. New research lines must be developed to prevent/treat obesity and hence the dysregulation of peptides exerting an orexigenic (obesity) or an anorexigenic (leanness) effect must be investigated in-depth to understand the mechanisms involved in feeding behaviour. GAL, by inhibiting orexin neurons, acts as a mediator of the leptin action to regulate nutrient reward. It is clear that emotional/reinforcement factors are involved in feeding behaviour and, for this reason, extra-hypothalamic regions which play an important role in these factors must be also investigated to select new potential therapeutic targets. In this sense, the release of dopamine promoted by GAL into the nucleus accumbens shell is involved, not in the consumption of fat, but in the motivational processes regulating such consumption. The involvement of the GAL 1 receptor in motivation at times of high appetitive behavior must be also investigated as well as the role played by GAL, via interaction with norepinephrine, in stress-induced overconsumption. GAL mediates fat consumption via the GAL 1 receptor; however, the involvement of GAL 2 and 3 receptors in feeding mechanisms must be elucidated since, for example, at hypothalamic level, the GAL 2 receptor plays an important role in the ingestion of palatable foods. Research focused on the involvement of alarin, GAL-like peptide and GAL message-associated peptide in feeding behaviour must be potentiated. For example, it is important to demonstrate the existence of native receptors for these three peptides. This is crucial to know in detail the anti-obesity action mediated by GAL-like peptide and its transport through the blood–brain barrier. In feeding mechanisms, it is also important to know the upregulation/downregulation of endopeptidases (e.g., GAL is a substrate of neprilysin), since upregulation of the endopeptidase neprilysin can decrease the level of body fat. The roles played by GAL-like peptide and GAL 2 receptor agonists as antidiabetic agents for the treatment of diabetes (type 2)/insulin resistance must be investigated and confirmed, and the role played by the neurosecretory protein GL in feeding behaviour must be fully elucidated. Another important topic is to know how the different fragments of the GAL family of peptides (e.g., GAL_1-15_, alarin 6-25Cys) are involved in feeding behaviour exerting orexigenic or anorexigenic actions and the physiological interactions between these fragments must also be investigated. The role of GAL as a neuroprotective and/or neurotrophic agent in the protection of the enteric nervous system neurons against the harmful effect mediated by acrylamide must be demonstrated, as well as the mechanisms mediated by bisphenol A regarding the alterations observed in sympathetic fibers containing GAL. It is important to note that a low dose of acrylamide promoted neurochemical changes in peripheral nerve cells; thus, according to the results published it seems that the level of acrylamide must be drastically reduced in food products to avoid these changes. Moreover, it must be fully demonstrated whether GAL, after a chronic intake of fat, counteracts the harmful effects mediated by inflammatory cytokines. It must be clearly determined whether obesity increases the secretion of GAL and whether the GAL serum level could serve as a biomarker for the prediction of impaired glucose tolerance. Moreover, research on SNPs associated with obesity must be developed. Heteromers (GAL 1 receptor/µ-opioid receptor) have been suggested as targets for the treatment of food intake control loss. Studies focusing specifically on GAL receptor/NPY Y_1_ receptor interactions in hypothalamic and extra-hypothalamic nuclei are important since GAL blocks the food intake mediated by NPY.

Sexual differences have been reported regarding the involvement of some hypothalamic regions (e.g., medial preoptic nucleus) in fat intake and regarding the expression of GAL. This is also an important research line that must be developed in the future, since the level of GAL has been positively correlated with body fat (adiposity/obesity) and it seems that female puberty onset is mediated by steroids which stimulate GAL to increase fat intake. It has been reported that fatty acids, arising from the hydrolysis of the circulating triglycerides, can cross the blood–brain barrier and exert a central action by controlling the expression of enkephalin. In the case of GAL, this remains to be demonstrated. Regarding fat metabolism, it is important to know the molecular mechanisms, intracellular pathways and GAL receptors involved in the opposite effects mediated by spexin and GAL. GAL plays an important role in insulin/glucose metabolism. In this sense, it is important to study in-depth the possible GAL resistance in obese individuals to better understand the molecular mechanisms by which GAL regulates insulin/glucose metabolism. GAL does not exert a pivotal role in weight regulation and food intake, but this role is crucial in fat intake and also plays an important role in regulating the activity of other key compounds under conditions of stress/altered diet.

In sum, the involvement of the GAL family of peptides in feeding behaviour is reviewed. Here, the mechanisms of action and physiological roles played by this family of peptides in the control of food intake are mentioned as well as the involvement of these peptides in metabolic diseases and feeding disorders in experimental animal models and humans. The interaction between GAL and NPY in feeding and energy metabolism, the relationships between GAL and other substances involved in food intake mechanisms, the potential pharmacological strategies to treat food intake disorders and obesity and the possible clinical applications are discussed. Finally, some research lines that must be developed in the future are also suggested.

## Figures and Tables

**Table 1:** GAL family of peptides and food intake: general findings [2,38].

**General characteristics of GAL:** - fat intake via GAL 1 receptor - level correlated with amount of consumed fat; high level in the middle of the feeding cycle when fat preference is increased - positive feedback loop facilitating an excessive fat intake (obesity) - no effect on preference for fat over carbohydrates/proteins - feeding responses more prolonged/stronger with a high-fat diet
**Effects of GAL mediated by PVH:** - increase of GAL level when fat preference occurred - activation of GAL 1 receptor increased adiposity, food intake, body weight - GAL antagonists and reduced GAL level decreased fat intake
**Effects of GAL-like peptide:** - orexigenic: mediated by dopamine, orexin, NPY - anorexigenic: mediated by interleukins-1α and 1β actions

**Table 2:** Experimental animal models: GAL family of peptides and food intake [2,26,49,50,54,55,57,58,63,66,67,69,70,73,74,75,77,78,79,80,81,82].

Rat
Animal model of type 2 diabetes mellitus: - lower GAL plasma levels than controls - GAL blocks insulin release, accelerates glucose transport into cells increasing insulin sensitivity and glucose intolerance - GAL increases body weight, food intake, glucose transporter 4 mRNA expression and mitigates insulin resistance via GAL 2 receptor Genetically obese model: - hypothalamic GAL expression enhanced; GAL (GAL 1 receptor) decreased motivation at times of high appetitive behaviour Animal model of hyperphagia: - GAL gene expression upregulated, hyperresponsive to appetite-stimulated actions after GAL administration Dietary intervention-acute high fat diet: - cognitive deficits, GAL transcript level increased, GAL 1 receptor level decreased Animals after administration of daidzein: - food intake reduction by blocking GAL/NPY and by increasing CRH expressions Actions of GAL-like peptide in rat models: - decrease of energy input acting at the level of the nucleus of the solitary tract, hypothalamic control of the body weight, increased energy expenditure - administration into PVH: increase in food intake - administration into the ventricle/nucleus of the solitary tract: decrease in food intake - animal model of type 2 diabetes mellitus: blocked diabetes development and induced insulin resistance; gene expression regulated by insulin/glucose circulating levels. GAL-like peptide level decreased and returned to normal values after insulin/leptin administration
**Mouse**
Genetically modified models: - GAL-knockout animals: consumed less fat than controls (fat only available) - GAL-overexpressing animals: consumed more fat than controls - homozygous GAL transgenic animals: higher GAL serum level, increase in visceral adiposity, body weight and triglycerides/cholesterol serum levels; energy expenditure decreased Dietary interventions: - high fat diet: control animals gained more body weight than GAL-knockout animals - hedonically-loaded foods blocked the inhibitory actions of leptin on orexin neurons; GAL is a mediator of leptin action regulating nutrient reward by inhibiting orexin neurons - overeating palatable foods: decreased in lean mice by activating the GAL 2 receptor (lateral hypothalamus) - administration of green tea extract: decreased food intake, body fat/weight, prevented fat accumulation, increased peripheral activity/expression of neprilysin (downregulation of GAL, NPY) - diet-induced obese animals treated with celastrol: decreased obesity, food/fat intake, induced weight (suppressing expression of GAL/GAL receptors; anti-obesity action) Mouse models with mixed pathologies: - underweight animals with signs of anxiety, depression and anhedonia: GAL 2 receptor agonists normalized body weight/mood behaviours. Hypothalamic CRH/POMC and dorsal raphe serotonergic neurons involved Actions of GAL-like peptide in mouse models: - GAL-like peptide knockout animals: gained less weight after administration of a rich fat diet - animals with a diet-induced obesity and administered with GAL-like peptide: inhibition of lipid accumulation in liver, body weight decreased - intranasal administration of GAL-like peptide: effective route to exert an anti-obesity action - spontaneously exercising animals: GAL-like peptide favoured energy metabolism; heat production level/consumed oxygen volume increased; body weight decreased. - under altered dietary situations, GAL-like peptide maintained the metabolic homeostasis; its involvement in energy balance mechanisms was not pivotal - fasting or hyperglycemia respectively downregulated or upregulated transport of GAL-like peptide through the blood–brain barrier Actions of alarin in mouse models: - increased food intake, body weight and luteinizing hormone plasma level
**Pig**
Animal models for toxicity studies: - administration of acrylamide: increased the number of immunoreactive neurons containing GAL and increased GAL-immunoreactivity in the stomach enteric nervous system neurons. GAL can protect the enteric nervous system neurons against the harmful effect mediated by acrylamide. GAL could play a neuroprotective and/or neurotrophic action - administration of bisphenol A: changed the expression of GAL in the duodenum enteric nervous system/intrahepatic sympathetic fibers

**Table 3:** Humans: GAL family of peptides and food intake [2,7,33,38,56,83,86,87,89,90].

**Obesity-related pathologies:** - obesity increased GAL secretion - women with moderate/severe obesity: high plasma concentration of GAL - GAL plasma level higher in fat women than in thin ones - obese women with anorexia nervosa: higher GAL plasma level than control women - obese menopausal women: high GAL serum level. Recovered anorexic women: GAL level decreased in cerebrospinal fluid
**Obese children/adolescents:** - children: GAL associated with lipid metabolism/glucose homeostasis - children: higher leptin/GAL serum levels than in healthy subjects. GAL level positively correlated with insulin level and triglycerides/insulin resistance - children/adolescents: no association between GAL 2 receptor/obese phenotypes - children/adolescents: no allelic difference in GAL 1 receptor or GAL compared to controls
**Diabetes/glucose level pathologies:** - healthy subjects/those suffering from type 2 diabetes: positive relationship between blood glucose/GAL levels - non-pregnant women with diabetes: increased GAL plasma level - pregnant women with gestational diabetes mellitus: higher circulating GAL level; positive correlation between glucose/GAL levels - patients with impaired glucose tolerance: negative correlation between glucose/GAL levels - GAL serum level: potential biomarker for the prediction of impaired glucose tolerance
**Alarin levels:** - higher in metabolic syndrome patients than in healthy subjects - circulating alarin level: positively correlated with blood pressure, fasting blood glucose, AUCglucose, glycated haemoglobin, homeostasis model assessment of insulin resistance, triglyceride and tumor necrosis factor α - high circulating alarin level: associated with metabolic syndrome/insulin resistance
**Administration of Spexin (anti-obesity agent):** - decreased body weight/blocked food intake. Actions exerted on adipocyte metabolism mediated by GAL 2/3 receptors - blocked adipogenesis, downregulated mRNA expression of pro-adipogenic genes
No SNPs of GAL have been fully associated with obesity or altered body mass index

**Table 4:** GAL and other substances: relationships [2,26,38,55,91,92,93,94,95,96,97,98,99].

Dopamine
- GAL increases feeding intake by increasing dopamine level in the nucleus accumbens - Dopamine release (mediated by GAL) into the nucleus accumbens involved in the motivational processes regulating fat consumption
**Norepinephrine**
- GAL orexigenic effect: due to activation of GAL 1 receptor /5-HT_1A_ receptor heteromers which blocked 5-HT_1A_ activation/increased food consumption - GAL (into central nucleus of the amygdala): food intake mediated by an interaction with norepinephrine. GAL mediated stress-induced overconsumption - PVH nucleus: feeding mechanisms mediated by GAL due to effects promoted by norepinephrine - Satiated rodents: norepinephrine promoted food intake when administered into PVH - Feeding mechanisms induced by GAL: mediated by noradrenergic alpha-2/5-HT_1A_ receptors
**Epinephrine**
- Epinephrine blocked starvation-induced secretion of GAL
**Enkephalin**
- PVH nucleus: GAL interacted with enkephalin; fat ingestion promoted the expression of both peptides; µ-opioid receptor antagonists blocked fat ingestion mediated by GAL
**Beta-endorphin**
- Food intake mediated by GAL: due to the release of beta-endorphin controlled by GAL
**Cocaine- and amphetamine-regulated transcript peptide**
- Enteric nervous system: co-existence of CART/GAL
**Enterostatin**
- Enterostatin blocked the stimulated feeding action mediated by GAL
**Insulin**
- PVH nucleus: positive relationship between GAL gene expression, amount of fat ingested and blood glucose level - PVH nucleus: GAL administration decreased insulin level - High-fat diet: insulin decreased GAL gene expression (PVH); a decrease in insulin level promoted GAL overexpression (PVH) - Diabetic rats: insulin detemir reduced food intake/weight gain by downregulating hypothalamic GAL mRNA/protein expressions - Dark/light feeding cycle: inverse relationship between insulin and PVH GAL level
**Leptin**
- GAL blocked sensitivity of fat tissue/body weight to leptin and reduced synthesis/secretion of leptin in the visceral adipose tissue - PVH nucleus: leptin decreased body weight/eating behaviour and GAL expression; leptin regulated fat ingestion
**Growth hormone**
- Growth hormone (into hypothalamus): decreased hypothalamic GAL level, energy expenditure and lipid oxidation
**Progesterone**
- Progesterone: involved in the development of obesity in females with a high-fat diet consumption - Female puberty onset is mediated by steroids which stimulate GAL to increase fat intake - PVH nucleus: positive relationship (proestrous period) between GAL level/circulating progesterone - Medial preoptic nucleus: progesterone increased GAL expression in this nucleus in females; involved in fat intake in females, but not in males
**Cytokines**
- Hypothalamus: chronic administration of a fat-rich diet increased tumor necrosis factor-alpha/interleukin 1-beta level which stimulate GAL gene expression - Chronic intake of fat: GAL counteracted the harmful effects mediated by inflammatory cytokines
**Triglycerides**
- GAL overexpression: higher level of circulating triglycerides - PVH nucleus: GAL is related to saturated fatty acids intake; GAL expression is positively related to circulating level of triglylcerides; intralipid administration in this nucleus increases GAL expression and triglylcerides circulating level
**Neuropeptide Y**
- GAL promotes the secretion of NPY by hypothalamic neurons - GAL inhibits food consumption mediated by NPY Y_1_ receptor agonists - Nerve cells expressing NPY or GAL show synaptic contacts with each other in hypothalamic nuclei: functional GAL/NPY interactions occur in feeding behaviour and energy metabolism - Arc: GAL receptor interacts with NPY Y_1_ receptor and decreases binding of NPY Y_1_ receptor agonists; effect counteracted by M-35 (GAL receptor antagonist) - Arc: GAL inhibits food intake mediated by NPY Y_1_ receptor agonists through a GAL receptor–NPY Y_1_ receptor interaction - Arc: NPY neurons send inhibitory inputs to GAL neurons located in PVH - Arc: carbohydrate intake, regulated by NPY, decreases fat intake by the inhibition exerted by NPY on GAL neurons
